# Database of adverse events associated with drugs and drug combinations

**DOI:** 10.1038/s41598-019-56525-5

**Published:** 2019-12-27

**Authors:** Aleksandar Poleksic, Lei Xie

**Affiliations:** 1 Department of Computer Science, University of Northern Iowa, Cedar Falls, Iowa 50614 USA; 2 Department of Computer Science, Hunter College, The City University of New York, New York, New York 10065 USA; 3 Ph.D. Program in Computer Science, Biochemistry and Biology, The Graduate Center, The City University of New York, New York, New York 10065 USA

**Keywords:** Drug safety, Molecular medicine

## Abstract

Due to the aging world population and increasing trend in clinical practice to treat patients with multiple drugs, adverse events (AEs) are becoming a major challenge in drug discovery and public health. In particular, identifying AEs caused by drug combinations remains a challenging task. Clinical trials typically focus on individual drugs rather than drug combinations and animal models are unreliable. An added difficulty is the combinatorial explosion in the number of possible combinations that can be made using the increasingly large set of FDA approved chemicals. We present a statistical and computational technique for identifying AEs caused by two-drug combinations. Taking advantage of the large and increasing data deposited in FDA’s postmarketing reports, we demonstrate that the task of predicting AEs for 2-drug combinations is amenable to the Likelihood Ratio Test (LRT). Our pAERS database constructed with LRT contains almost 77 thousand associations between pairs of drugs and corresponding AEs caused solely by drug-drug interactions (DDIs). The DDIs stored in pAERS complement the existing data sets. Due to our stringent statistical test, we expect many of the associations in pAERS to be unrecorded or poorly documented in the literature.

## Introduction

An adverse event (AE) is an injury caused by a drug. Adverse events account for more than 100,000 deaths per year in the United States^1^, representing the 4^th^ leading cause of death, after heart disease, cancer and stroke, but well ahead of diabetes, pulmonary diseases and AIDS.

Due to limited duration and small patient population, clinical trials lack the necessary statistical power to identify severe AEs. To address this limitation, postmarketing safety reporting was established (FAERS - https://open.fda.gov/data/faers). The assumption is that spontaneous reporting by health-care professionals, as well as consumers, family members and lawyers provides a more complete and accurate data on drug-AE associations. Nevertheless, the data from postmarketing safety surveillance is neither clean nor reliable. Part of the problem is that the adverse events in FAERS are reported cumulatively for all drugs taken by a patient, making it difficult to tell which drug, among multiple ones given to the patient, gave rise to the reported side-effects.

### AEs arising from drug combinations

While limited information on AEs of individual drugs is available in the drug package inserts and lately in electronic forms^2,3^, there is an utter lack of data on side-effects arising from drug-drug interactions. On the other hand, there is an increasing trend in clinical practice to treat patients with drug combinations. Almost 80% of Americans over the age of 60 are taking multiple drugs^4^, and the number of drugs taken by a patient is the most reliable predictor of the occurrence and intensity of an adverse event^5^.

There are two reasons behind the sparseness of data on side-effects arising from drug combinations. First, clinical trials typically focus on a single compound and rarely on drug combinations. Additionally, a combinatorial explosion occurs in the number of possible drug therapies that can be made using the current market drugs. For instance, the existing set of ~1,500 FDA approved drugs yields more than one million 2-drug combinations. Such an enormous number of combined therapies makes the task of studying, organizing and storing AEs computationally challenging.

Due to the above difficulties, the existing AE resources are scarce. Among noteworthy exceptions are SIDER^2,3^ and the OFFSIDES^6^ database of AEs for individual drugs, as well as DrugBank^7^ and the TWOSIDES^6^ databases of AEs for pairs of drugs. While drug-drug interactions (DDIs) in DrugBank mainly come from manual curation of literature, the statistical significance of associations in TWOSIDES is computed using Propensity Score Matching (PSM)^8,9^. In addition to statistical techniques, network-based multi-scale modeling may provide critical mechanistic insights into DDIs^10,11^.

Here we demonstrate that the problem of detecting adverse events caused by DDIs is amenable to Likelihood Ratio Test (LRT) statistics^12^. LRT has been originally developed to address problems in fields as diverse as astronomy, forestry, zoology and epidemiology. The theory has been successfully applied to the study of the sudden infant death syndrome (SIDS)^12^ as well as in cancer surveillance to find clusters of high disease incidence and mortality^13^. In a study most relevant to ours, Huang *et al*., have demonstrated that LRT can lower the type I error in detecting AEs associated with one-drug therapies^14^.

## Methods

We constructed two groups of two different AE databases. The first group contains associations between AEs and individual drugs, while the second group contains associations between AEs and drug combinations. In order to allow for more diverse user queries, we developed two databases for each group, one consisting of MedDRA Preferred Terms (PT)^15^ and one where AEs represent MedDRA High Level Terms (HLT).

### Normalizing drug and AE names stored in FAERS reports

#### Drug lexicon

We use FDA’s Orange Book (https://www.accessdata.fda.gov/scripts/cder/ob/) to compile a set of representative drugs. To remove duplicate drugs (those that contain the same ingredient but in different pharmaceutical salt forms) we use RxNorm^16^- a suite of databases and tools that provides normalized names for clinical drugs. As of this writing, applying those filters yields a set of 1,309 unique chemicals. In order to boost the statistical power in extracting AEs for highly similar drugs and their combinations, we use the Complete-linkage clustering (Statistical Machine Intelligence and Learning Engine, http://haifengl.github.io/smile/) to group 1,309 drugs into 1,178 clusters. We define the dissimilarity of (i.e., the distance between) compounds *c*_1_ and *c*_2_ as the “inverse” Tanimoto metric^17^, namely, $dist({c}_{1},{c}_{2})=1-Tanimoto({c}_{1},{c}_{2})$. We note that the Complete-linkage requires no parameters and is preferable to both Single-linkage and Average-linkage clustering since it is capable of finding compact clusters of approximately equal diameters^18^.

#### AE vocabulary

In contrast to normalizing drugs, normalizing AE names is a relatively simple task, because, by default, FAERS reports use MedDRA “preferred terms” (PTs) for adverse events that appear under the “reactionmeddrapt” field. Since FAERS safety reports always employ the most up-to-date release of MedDRA for ADR lexicon, the ADR normalization task amounts to mapping “preferred terms” (PTs) that appear in older safety reports (in the “reactionmeddrapt” field) to corresponding PT terms from the latest MedDRA dictionary. We configured our parser to adopt to frequent (twice a year) releases of MedDRA in order to take advantage of data from as many safety reports as possible.

What makes our task somewhat easier is the fact that MedDRA updates follow a set of well-defined rules. Namely, each MedDRA release either preserves the “preferred terms” (PTs) or, less frequently, demotes them to “lowest level terms” (LLTs). Hence, mapping an old to new PT boils down to searching for the “demoted” PT in the set of LLTs from the most recent MedDRA database. Once such an LLT is found, a new name for the old PT is assigned by moving up one edge in MedDRA hierarchy. Following this approach, our algorithm is able to map 99.8% (34,520 out of 34,599) of all unique AE strings from the current FAERS set of patient reports to the corresponding AE terms in the most recent MedDRA dictionary (as of this writing, MedDRA 21.0). The remaining 79 strings are simply discarded as most of them represent arbitrary and often meaningless user entries.

### Extracting the set of representative FAERS reports

We downloaded a comprehensive set of 822 safety report files (all files available at FAERS as of March. 2019) containing over 9 million patient safety reports (Table 1). While LRT statistics reduces the selection bias by a large degree, it is important to preprocess raw data in order to dampen the effect of confounding variables, such as therapeutic indications (TIs). Our goal is to discard any drug-AE pair (or, in case of drug combinations, any therapy-AE pair) in which the AE represents a known therapeutic indication for the drug (respectively, one of the two drugs from the combination therapy). For instance, deep vein thrombosis (DVT) often appears as an AE in a safety report for a patient that takes an anticoagulant for DVT or related disease or condition (e.g., pulmonary embolism). However, DVT represents the condition/disease rather than an AE in majority of cases where the list of drugs taken by the patient includes an anticoagulant. For this reason, we discard all therapy-AE pairs where the AE represents a therapeutic indication for one or both drugs comprising the therapy.

**Table 1:** Preprocessing patient reports.

Action	# reports	
download 822 FAERS files	9,487,279	
normalize drugs, AEs & remove TIs	4,848,332 (PT)	3,304,130 (HLT)
removing duplicate reports	3,701,413 (PT)	2,323,193 (HLT)

Instead of parsing the drug package inserts, as done in SIDER, we compile a comprehensive set of indications for each of 1,309 drugs from our list by parsing the FAERS’ “drugindication” field. This process results in a set of drug-TI pairs along with the total number of occurrences of each pair in FAERS reports. For increased specificity, we discard extremely rare drug-TI pairs, those that appear less than a predefined number of times (default = 5) in the FAERS database. We enrich the remaining set of therapeutic indications using MedDRA’s PTs and LLTs. For instance, if a therapeutic indication for a particular drug represents a PT, the list of indications for that drug is expanded to include all LLTs found below that PT in MedDRA hierarchy. If a therapeutic indication represents a LLT, the drug indication set is expanded to include the “parent” PT. Finally, we remove all drug-AE pairs that represent drug-TI pairs.

We also implement a simple procedure for removing duplicate reports. We consider two reports the same if they list the same set of medications (specified in “medicinalproduct” field), the same set of adverse events (“reactionmeddrapt” field) and the same “patientonsetageunit”, ”patientonsetage”, “occurcountry”, “patientsex” and ”patientweight”.

### Compiling raw therapy-AE counts

A given safety report might list any number of medications and any number of adverse events. This data is given cumulatively for each patient without any reference to possible associations between drugs and AEs. While defining observed drug-AE counts is straightforward for individual drugs, it is less clear how to do this for drug combinations. Hypothetically, if the number of drugs taken by a patient was exactly two, it would make sense to say that such a drug combination contributes a single count for every AE in the report. A typical report, however, lists more than two drugs, making it difficult to tell which one of *k* = *C*(*m*, 2) combinations of two out of *m* drugs gave rise to each AE reported by the patient.

We adopt a simple approach to defining therapy-AE counts for 2-drug therapies. Assuming that each 2-drug combination is equally likely to cause each AE, a patient report that lists *m* drugs contributes 1/*C*(*m*,2) to every possible 2-drug therapy and each AE that appears in the report. Under this simple scheme, for instance, a report that contains exactly two drugs contributes one count to each AE listed in the report. If a report contains three drugs, the corresponding contribution is 1/3, etc. We use the sparse matrix representation (Linear Algebra for Java, http://la4j.org/) to circumvent large memory requirement arising from the enormous number of drug combinations and accompanying adverse events.

### Likelihood ratio test statistics (LRT) for therapy-AE associations

We follow the LRT procedure in Huang *et al*.^14^ to assign *p*-values to associations between individual (single) drugs and AEs. The same procedure is extended to assign *p*-values to raw therapy-AE counts for therapies consisting of two drugs. LRT allows us to detect adverse and safety signals from groups of ADRs or groups of drugs (or drug combinations) simultaneously, thereby adjusting for the effects of the key confounding variables on the fly. We employ the LRT statistics to solve a two-fold problem: (A) Given a 2-drug therapy, find AEs that have high reporting rates, compared to other AEs for the same 2-drug therapy, and (B) Given an AE, find all 2-drug therapies that have high reporting rates for the AE, compared to reporting rates of other 2-drug therapies. What this means in practice is that LRT is applied once for every 2-drug therapy and once for every AE under consideration. For every therapy-AE pair, the larger of the two *p*-values (therapy-specific and AE-specific) represents the statistical significance of the therapy-AE association. Below, we describe the algorithm for computing the therapy-specific *p*-values.

**Figure 1:**
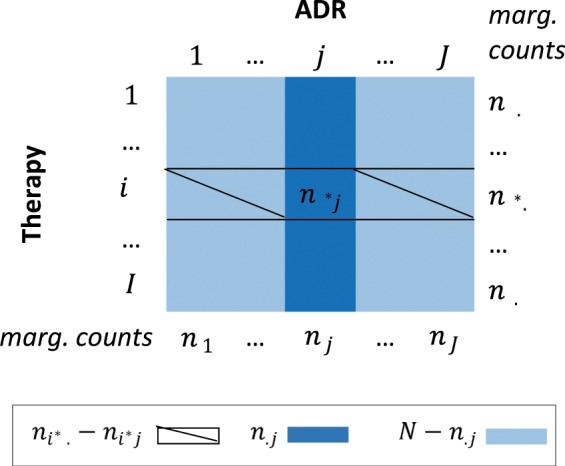
The components of LRT statistics. The matrix of raw therapy-AE counts.

Since each AE is a rare event, we assume that the number of times a drug combination i^*^ causes an AE *j* follows the Poisson distribution ${n}_{{i}^{\ast }j} \sim Poisson({n}_{.j}\times {p}_{{i}^{\ast }j})$, where ${p}_{{i}^{\ast }j}$ denotes the reporting rate of drug combination i^*^ for AE *j* and ${n}_{.j}$ is the marginal total for AE *j* i.e., ${n}_{.j}=\sum _{i}{n}_{ij}$ (Fig. 1). We also assume that the remaining AE counts for therapy i^*^ are Poisson distributed i.e.,

$({n}_{{i}^{\ast }.}-{n}_{{i}^{\ast }j}) \sim Poisson(N-{n}_{.j}\times {q}_{{i}^{\ast }j})$, where ${n}_{{i}^{\ast }.}=\sum _{j}{n}_{{i}^{\ast }j}$, ${q}_{{i}^{\ast }j}$ represents the reporting rate of drug combination i^*^ for all other AEs (excluding *j*-th AE), and *N* is the total number of observations, $N=\sum _{i,j}{n}_{ij}$. The null hypothesis is that the reporting rates for all AEs are the same i.e., ${p}_{{i}^{\ast }j}={q}_{{i}^{\ast }j}={p}_{{i}^{\ast }0}$, for all *j*. The two-sided alternative is that ${p}_{{i}^{\ast }j}\ne {q}_{{i}^{\ast }j}$, for at least one AE *j*. Following Huang *et al*.^14^, we define the test statistics as $MLR=\mathop{\max }\limits_{j}L{R}_{{i}^{\ast }j}$, where $L{R}_{{i}^{\ast }j}$ represents the likelihood ratio for drug combination ${i}^{\ast }$ and AE $j$, namely

$$L{R}_{{i}^{\ast }j}=\frac{{\hat{p}}_{{i}^{\ast }j}^{{n}_{{i}^{\ast }j}}{\hat{q}}_{{i}^{\ast }j}^{{n}_{{i}^{\ast }.}-{n}_{{i}^{\ast }j}}}{{{\hat{p}}_{{i}^{\ast }0}}^{{n}_{{i}^{\ast }.}}}$$

where ${\hat{p}}_{{i}^{\ast }j}=\frac{{n}_{{i}^{\ast }j}}{{n}_{.j}}$, ${\hat{q}}_{\bar{i}j}=\frac{{n}_{{i}^{\ast }.}-{n}_{{i}^{\ast }j}}{N-{n}_{.j}}$ and ${\hat{p}}_{{i}^{\ast }0}=\frac{{n}_{{i}^{\ast }.}}{N}$ are the maximum likelihood estimates of ${p}_{{i}^{\ast }j}$, ${q}_{{i}^{\ast }j}$, and ${p}_{{i}^{\ast }0}$, respectively.

Since the joint distribution of $({n}_{{i}^{\ast }1},\ldots ,{n}_{{i}^{\ast }J})$ under the null hypothesis is multinomial^14^

$$({n}_{{i}^{\ast }1},\ldots ,{n}_{{i}^{\ast }J})|{n}_{{i}^{\ast }.;}{n}_{.1},\ldots ,{n}_{.J} \sim mult({n}_{{i}^{\ast }.;}\frac{{n}_{.1}}{N},\ldots ,\frac{{n}_{.J}}{N}),$$

the background distribution of $MLRs$ for ${i}^{\ast }$ can be obtained by simulating a number of datasets from the above distribution. We use parallel algorithm implementations to deal with computational expense in generating the background distributions. We reject the null hypothesis at *α* level if the *MLR* from the observed raw counts ${n}_{{i}^{\ast }1},\ldots ,{n}_{{i}^{\ast }J}$ exceeds a predefined percentage (e.g. 99%, corresponding to *α* = 0.01) of *MLRs* calculated from Monte Carlo simulations. The alpha level balances the likelihood of Type I and Type II errors. The commonly accepted alpha levels are between 0.01 and 0.05. In our case, a more conservative *α* = 0.01 is selected to address the potentially high rates of false positives that are due to differential reporting in FAERS. Once the strongest AE signal for the 2-drug combination i^*^ is detected, other strong AE signals for the same therapy are found by moving down to the second largest value of the likelihood ratio test statistics $L{R}_{{i}^{\ast }j}$ and testing the null hypothesis again. The *p*-values assigned to each MLR score represents the rank of that score in the set of all background MLR scores calculated from Monte Carlo simulations^14^.

For drug combination i^*^, the above procedure will accomplish the first task (task A), namely, it will detect ADRs that have high reporting rates for i^*^ compared to other ADRs. The task of finding ADRs that have a high reporting rate for a given drug combination compared to other drug combinations (task B) is accomplished by reversing the roles ADRs and drug combinations e.g., by swapping rows and columns of the count matrix (*n*_*ij*_).

## Results

### Database statistics

**Figure 2:**
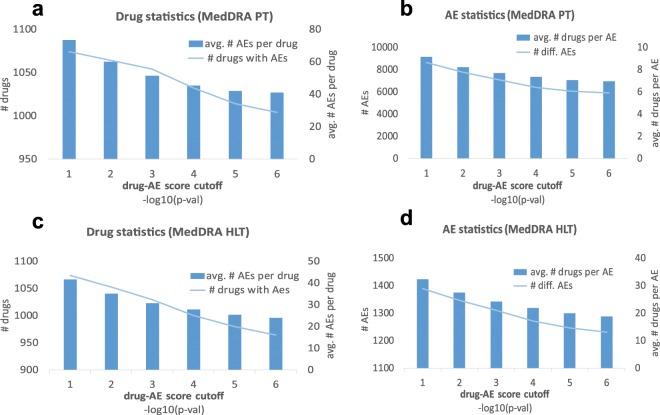
Cumulative and average statistics for AEs associated with single drug therapies. For each drug-AE *p*-value cutoff, we show the cumulative number of drugs and AEs (left axes in **a,c** and **b,d** respectively). We also give the average number of AEs per drug and the average number of drugs per AE (right axes in **a,c** and **b,d**, respectively).

**Figure 3:**
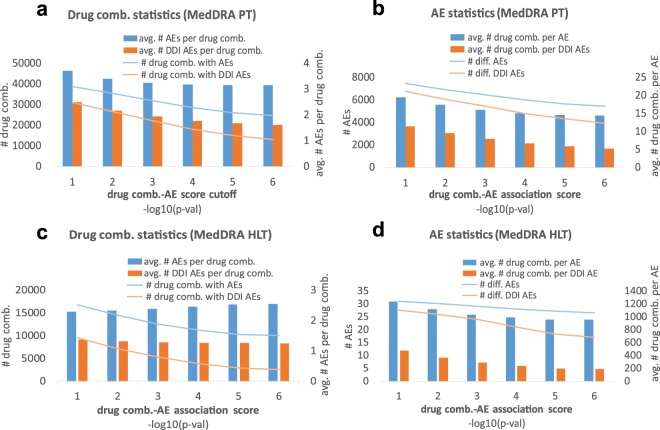
Cumulative and average statistics of therapy-AE associations for 2-drug therapies. For each therapy-AE *p*-value cutoff, we show the cumulative number of two-drug therapies and AEs (left axes in **a,c** and **b,d** respectively). We also give the average number of AEs per therapy and the average number of therapies per AE (right axes in subgraphs **a,c** and **b,d**, respectively). The DDI statistics (computed after removing AEs associated with either drug from the therapy) is shown in red.

Table 2 and Figs. 2 and 3 show the summary statistics for our pAERS databases. To view this data in context, we note that the total number of PT and HLT terms in MedDRA 21.0 is 23,088 and 1,737 respectively, while the number of possible therapies (2-drug combinations) that can be made out of 1,178 drug clusters is 693,253.

**Table 2:** pAERS statistics. Data on AEs unique to drug interactions in parentheses.

	Individual drugs		Drug combinations	
	*PT*	*HLT*	*PT*	*HLT*
# therapies	1,074	1,074	38,842 (30,872)	16,754 (9,586)
#AEs	8.167	1,389	7,451 (6,736)	1,238 (1,107)
# entries	78,686	44,743	144,335 (76,902)	38,254 (13,074)
sparsity	8.5E-3	3.0E-2	5.0E-4 (3.7E-4)	1.8E-3 (1.2E-3)

#### Limitations and advantages of pAERS

Methods for detecting AE signals from postmarketing safety reports, such as LRT, face some common obstacles. If a drug (or one or both drugs from a 2-drug therapy) is relatively new, the data on said drug (or drug combination) reported in FAERS will be sparse, thereby reducing the statistical power of LRT. The same is true for drug combinations that are known to cause harmful AEs. Those combinations are rarely prescribed by physicians and, in turn, are poorly documented in FAERS. Taking all the above into account, the statistically insignificant therapy-AE associations in pAERS should be viewed as missing (rather than negative) data. The missing entries in pAERS can be recovered using techniques such as compressed sensing^19,20^ and collaborative filtering^21,22^ but those methodologies are outside of the scope of present study.

On the flipside (as we demonstrate later on the *cyclosporine* interactions) we expect many associations recorded in pAERS to be poorly documented in the literature. In order for LRT to declare a therapy-AE association “significant”, the association must pass two tests: the therapy must have a high reporting rate for AE and vice versa. Even innocuous and very frequent AEs (e.g., nausea or dizziness) will not show up in our database unless their reporting rates exceed the background reporting rates. Due to such stringent requirements, the LRT statistics have been shown to lower the Type I error in detecting AE signals significantly more than alternative approaches^14^.

The low false discovery rate of LRT explains why the average numbers of AEs per therapy (Figs. 2 and 3) are below the corresponding figures in SIDER^2,3^, OFFSIDES^6^ and TWOSIDES^6^. Even the maximum number of AEs per drug in pAERS are “only” 484 and 365 for PT and HLT lexicon, respectively, while the corresponding figures for drug combinations are 378 and 117 (125 and 29 for AEs caused by DDIs).

We note that some false positives in pAERS are difficult to eliminate. For instance, pAERS reports a high rate of rheumatoid arthritis and related “AEs” resulting from the therapy consisting of anticoagulants and *tylenol*. This therapy is given to patients suffering from both blood coagulative disorders and rheumatoid arthritis, since *acetaminophen* represents a relatively safe way to managing pain in patients who are taking *warfarin* or other anticoagulants. The false positives of this type “sneak through” our algorithm in part because they cannot be removed as therapeutic indications (in our example, rheumatoid arthritis is not the indication for either type of drug).

#### Comparison to TWOSIDES database

Several methods have been published that aim to predict DDI induced adverse events^23,24^. Our method is different in spirit, as it seeks to extract (rather than predict with machine learning) data on adverse events. Previously, an algorithm based on Propensity Score Matching (PSM)^8,9^, had been used to develop the TWOSIDES database of AEs associated with pairs of drugs^6^. The authors of TWOSIDES demonstrate that PSM is capable of filtering out about half of synthetic associations between drugs and AEs.

With 6,602 unique AEs reported for all 2-drug therapies combined, pAERS_PT database is richer than TWOSIDES in the number of adverse events reported overall, but the number of AEs in pAERS_HLT tails 1,301 AEs in TWOSIDES. On the other hand, TWOSIDES is almost twice as large in the total number of pairs of drugs (59,220), while the number of reported associations in TWOSIDES (868,221) far exceeds the corresponding figure in pAERS.

A theoretical advantage of the PSM method used in TWOSIDES is that it controls for all variables that have effects on treatment outcome. However, the critiques of PSM approach argue that, by excessively pruning important data, PSM can, in fact, increase data imbalance and bias, leading to the so-called “PSM paradox”^25^. In contrast, the Likelihood Ratio Test has a provable low false discovery rate^12,14^ and it places all database *p*-values on a uniform ground. Just like PSM, LRT can efficiently correct for the selection bias and sampling variance. Moreover, LRT can take gender and age into account with the potential of personalized AE stratification. It should also be noted that pAERS provides for much better coverage of FDA approved drugs (66% more individual drugs than in TWOSIDES).

### Illustrative example - *Cyclosporine* interactions

We demonstrate the accuracy of our data extraction algorithm on the example of an immunosuppressant drug *cyclosporine* (*neoral*, *sandimmune*), which is commonly used to prevent rejection of liver, heart, lung, pancreas or bone marrow transplants.

In each of the examples below, the association between the drug pair and AE is due to DDIs, as evidenced by the LRT *p*-value ≤10^−6^ and a complete lack of association between the AE and any individual drug from the drug pair (*p*-value > 0.1).

#### Cyclosporine and azole antifungals

Immunocompromised patients, such as those receiving organ transplants, are affected by mycotic disease. In order to prevent infection and transplant rejection, *cyclosporine* is routinely co-administered with *antifungal azoles*^26^. However, interactions with azoles alter the concentrations of cyclosporine, increasing the risk of transplant failure. As seen in Table 3, LRT is able to detect statistically significant interactions between *cyclosporine* and different *azoles*, including *itraconazole, voriconazole, pantoprazole and secnidazole*.

**Table 3:** Cyclosporine interactions in pAERS.

Interacting Drug	Adverse Reaction
Itraconazole	Cardiac murmur
Pantoprazole	Osteomyelitis
Voriconazole	Tremor
Secnidazole	Atrial flutter, Palmar-Plantar erythrodysaesthesia
Rapamycin	Globulins decreased, Jacod syndrome
Prednisolone, Medrol	Colectomy, Peliosis hepatis (HLT)
Rosuvastatin	Hyperlipasaemia
Tecfidera or Methotrexate	Malignant melanoma stage I
Diprivan	Hyporeflexia, Mitochondrial myopathy acquired
Etoposide	Ocular vascular disorders NEX (HLT)

#### Cyclosporine and rapamycin

When co-administered with antifungal antibiotic *rapamycin*, *cyclosporine* is known to cause nephrotoxic injuries. We observe significantly *decreased globulin level* with *cyclosporine/rapamycin* therapy, demonstrating additional evidence of the reduction in renal function^27,28^ due to interaction of these two drugs.

#### Cyclosporine with prednisolone or medrol

*Cyclosporine* interactions with corticosteroids have long been recognized and recorded with numerous case studies documented in the literature^29,30^. In our database, these interactions manifest as severe AEs resulting from the interaction of *cyclosporine* with *prednisolone*. There is also a strong association of *Peliosis hepatis* (PH) with *cyclosporine/medrol* therapy in pAERS, confirming previous findings that the combination of these two drugs is the main cause of PH after liver transplantation^31^.

#### Cyclosporine and rosuvastatin

We identify a link between *cyclosporine/rosuvastatin* therapy and *high lipid levels*, confirming the relatively recent findings on interactions between the two drugs^32^. We observe no individual, significant *cyclosporine*-*hyperlipasaemia or rosuvastatin- hyperlipasaemia* associations.

#### Cyclosporine and tecfidera or methotrexate

*Cyclosporine* is known to cause skin cancer when used with other immunosuppressive agents^33^. We find *malignant melanoma* to be strongly associated with the *cyclosporine/methotrexate* therapy. The same adverse event appears in co-administration of *cyclosporine* with *tecfidera*, which has been known as a “safer” immunosuppressant agent.

#### Cyclosporine and diprivan

Enhanced toxic reactions are documented in patients taking *cyclosporine* with *propofol*^34^. In our database this interaction is manifested by high incidence of *hyporeflexia* and *mitochondrial myopathy*.

#### Cyclosporine and etoposide

The effect of *cyclosporine* treatment on etoposide PK has been previously reported in both adults^35^ and children^36^. We note statistically significant link between *cyclosporine/etoposide* therapy and the *ocular vascular disorders*.

The examples above demonstrate the robustness of LRT, compared to the statistical procedure used in TWOSIDES. In TWOSIDES, cyclosporine interacts with 60% of all other drugs, yielding a total of 27,671 adverse events combined (or 72 AEs per drug pair). In general, one in every three (30.4%) 2-drug therapies in TWOSIDES gives rise to at least one AE (with 73 AEs per therapy on average). This is, to say the least, unrealistic, in particular given that 94% of all *p*-values in TWOSIDES are below 1E-05.

While assessing the accuracy of our platform on a large scale is computationally prohibitive, studying individual drug pairs provides useful insight into the value added by our method. In another illustrative example, we searched for drugs interacting with *bosentan*, a dual endothelin receptor antagonist, commonly prescribed in the treatment of pulmonary artery hypertension. We found a significant probability of interactions between *bosentan* and *sildenafil*, *warfarin*, *norgestrel*, *ranolazine* and *clorazepate*, resulting in adverse events that cannot be contributed to any single drug from the combination. These findings confirm previously recorded interactions. For instance, multiple studies have been published documenting pharmacokinetic interaction between *sildenafil* and *bosentan* that may influence the dosage of each drug when co-prescribed in pulmonary hypertension^37,38^. Co-prescribing *warfarin* with *bosentan*, on the other hand, leads to increased risks to changes in International Normalized Ratios (INR), as demonstrated in different studies and individual case reports^39,40^. Furthermore, we observed an elevated risk of *neonatal respiratory distress syndrome*, in cases where *bosentan* was taken along with *norgestrel*, thereby confirming the interactions of *bosentan* with oral contraceptives recorded in the drug’s package insert and described in the literature^41^. Furthermore, we noted adverse events associated with co-prescriptions with CYP3A inhibitors and substrates, which are well known to decrease the excretion rate and serum concentration of the drugs co-prescribed with *bosentan*.

With the exception of *clorazepate*, the above interactions are also present in DrugBank^7^, but it should be noted that DrugBank is not informative enough to allow for a direct comparison. In DrugBank, the combined therapies with *bosentan* are only quoted to increase or decrease the serum level of the interacting drugs. A similar observation can be made for *cyclosporine* interactions in our first example. Moreover, the search with DrugBank’s Drug Interaction Lookup yields 1,135 and 2,192 drugs that can potentially interact with *bosentan* and *cyclosporine*, respectively. Such an enormous number of interacting drugs might be indicative of low specificity of DrugBank data.

Finally, we note that some associations in pAERS have not been previously observed and thus are difficult to assess and classify. For instance, it is difficult to say whether the supposed *bosentan*’s interactions with *orenitram* and *pregabalin*, stored in pAERS, represent novel discoveries or simply the Type I errors.

## Discussion and Conclusion

Nearly 4 out of 5 Americans over the age of 60 are taking multiple drugs^4^, while nearly one in three US adults are taking five or more medications^42^. Since the probability of an adverse event increases with the number of medications taken by a patient, predicting AEs is emerging as one of the most important tasks in drug discovery and biomedical research.

We present a statistical and computational method for accurately detecting adverse events arising from single- and two-drug therapies. While the therapy-AE associations stored in our databases need to be validated using epidemiological and clinical methods, the LRT technique adopted here has already been shown to reduce Type I error in detecting AE signals associated with single drug therapies.

The present study shares some common limitations with other predictive methods. The lack of statistical power of clinical trials makes it impossible to validate any individual association, let alone the millions of possible associations between all drug combinations and all adverse events. We are, however, aware of at least two limitations of our methodology. First, the LRT procedure lacks the statistical power in detecting interactions of new drugs due to insufficient postmarketing data on recently approved drugs. Second, our Monte Carlo simulation procedure used in estimating the association p-values is computationally expensive, due to the enormous number of drug combination and adverse events.

Better understanding of side-effects arising from drug interactions is becoming increasingly important, given a growing percentage of people who are prescribed multiple drugs and numerous scientific studies that show that the number of drugs taken by an individual is the most reliable predictor of an adverse event. Advances in computational prediction of adverse events arising from drug combinations will fuel the progress in drug repurposing and research in computational prediction of therapy-AE associations. We believe that our study is an important contributing step in this direction.

## Data Availability

The datasets generated during this study are available at https://gpubox.cs.uni.edu.
